# Platelet-to-Lymphocyte Ratio: A Novel Marker for Critical Limb Ischemia in Peripheral Arterial Occlusive Disease Patients

**DOI:** 10.1371/journal.pone.0067688

**Published:** 2013-07-02

**Authors:** Thomas Gary, Martin Pichler, Klara Belaj, Franz Hafner, Armin Gerger, Harald Froehlich, Philipp Eller, Peter Rief, Gerald Hackl, Ernst Pilger, Marianne Brodmann

**Affiliations:** 1 Division of Vascular Medicine, Department of Internal Medicine, Medical University Graz, Graz, Austria; 2 Division of Oncology, Department of Internal Medicine, Medical University Graz, Graz, Austria; Medical University Innsbruck, Austria

## Abstract

**Background:**

Platelet-to-Lymphocyte Ratio (PLR) is an easily applicable blood test. An elevated PLR has been associated with poor prognosis in patients with different oncologic disorder. As platelets play a key role in atherosclerosis and atherothrombosis, we investigated PLR and its association with critical limb ischemia (CLI) and other vascular endpoints in peripheral arterial occlusive disease (PAOD) patients.

**Methods and Findings:**

We evaluated 2121 PAOD patients treated at our institution from 2005 to 2010. PLR was calculated and the cohort was categorized into tertiles according to the PLR. An optimal cut-off value for the continuous PLR was calculated by applying a receiver operating curve analysis to discriminate between CLI and non-CLI. In our cohort occurrence of CLI significantly increased with an increase in PLR. As an optimal cut-off value, a PLR of 150 was identified. Two groups were categorized, one containing 1228 patients (PLR≤150) and a second group with 893 patients (PLR>150). CLI was more frequent in PLR>150 patients (410(45.9%)) compared to PLR≤150 patients (270(22.0%)) (p<0.001), as was prior myocardial infarction (51(5.7%) vs. 42(3.5%), p = 0.02). Regarding inflammatory parameters, C-reactive protein (median 7.0 mg/l (3.0–24.25) vs. median 5.0 mg/l (2.0–10.0)) and fibrinogen (median 457 mg/dl (359.0–583.0) vs. 372 mg/dl (317.25–455.75)) also significantly differed in the two patient groups (both p<0.001). Finally, a PLR>150 was associated with an OR of 1.9 (95%CI 1.7–2.1) for CLI even after adjustment for other well-established vascular risk factors.

**Conclusions:**

An increased PLR is significantly associated with patients at high risk for CLI and other cardiovascular endpoints. The PLR is a broadly available and cheap marker, which could be used to highlight patients at high risk for vascular endpoints.

## Introduction

Peripheral arterial occlusive disease (PAOD) is frequent and often not diagnosed in time [1]. If PAOD is not diagnosed and treatment is not initiated immediately, disease progression and development of critical limb ischemia (CLI) is one possible complication [2]. CLI is an entity with high mortality and high risk of limb amputation. Although treatment options, especially endovascular treatment possibilities, improved in the last decades, mortality and amputation rate are still high [3], [4].

In general, the ankle brachial index (ABI) can be used to distinguish CLI patients from non-CLI patients. However, the ABI might be unreliable due to mediasclerosis. In case of mediasclerosis the ABI does not reflect the perfusion in the extremity measured and therefore makes discrimination of CLI patients difficult. Especially in elder and diabetic patients – the patients with the highest CLI risk - mediasclerosis is frequently found [5].

In one recently published study we showed that a Neutrophil-to-Lymphocyte Ratio (NLR) >3.95 was associated with CLI as well as with other vascular endpoints [6]. Neutrophils included in this ratio reflect the inflammatory response as they mediate inflammation by various biochemical mechanisms, such as release of arachidonic acid metabolites and platelet-aggravating factors [7]. Relative lymphopenia on the other hand reflects the cortisol-induced stress response [7].

Platelet-to-Lymphocyte Ratio (PLR) is an easy to perform blood test associated with poor prognosis when elevated in patients suffering from various oncologic disorders [8], [9]. As platelets play a key role in atherosclerosis and atherothrombosis, we investigated PLR and its association with CLI and other vascular endpoints in peripheral arterial occlusive disease (PAOD) patients.

## Methods

We included 2121 consecutive PAOD patients treated at our department from 2005 to 2010 in our retrospective data analysis. Inclusion criterion for our analysis was treatment at our institution for PAOD during the time period described above. There was no exclusion criterion in our study. The study was approved by the International Review Board (IRB) of the Medical University of Graz, Austria (IRB Number 24–506 ex 11/12). As this was a retrospective data analysis of blinded data no written or verbal consent was obtained, which was approved by the ethics committee.

The diagnosis and graduation of PAOD was assigned in our outpatient clinic by means of clinical evaluation, ABI, and duplex scan according to the TASC II criteria. Patients were successive patients admitted to our outpatient clinic because of their PAOD and afterwards scheduled for admission at our ward for further treatment of their atherosclerotic disease. PAOD was graduated using Fontaine classification, CLI was defined as PAOD patients presenting with ischemic rest pain and/or skin ulceration/gangrene in accordance to current guidelines reflecting patients with Fontaine class 3 and 4 [10]. When patients were admitted to the hospital, the medical records of the patients were analyzed by a standardized questionnaire with attention to cardiovascular risk factors and co-morbidities. Clinical symptoms were evaluated and physical examination was performed. Blood was taken in fasting patients and laboratory examinations were performed. Total white blood cell count and differential counts were determined using automated analyser (Sysmex, Kobe, Japan).

### Statistical Analyses

Clinical characteristics of subjects were analyzed using descriptive statistics. For comparison of groups chi square test for categorical values, t-test for normally distributed continuous variables and Mann Whitney U test for non-normally distributed continuous variables were used.

The study population was divided into tertiles according to their continuous PLR. In order to reveal a statistical trend for PLR and CLI a Jonckheere-Terpstra test was performed. The optimal cut-off value for the continuous PLR was calculated by applying a receiver operating curve analysis to test all possible cut-offs that would discriminate between CLI and non-CLI.

We further calculated odds ratios (OR) with 95% confidence intervals (CI) for different CLI-risk factors with a binary logistic regression model. All tests used a p-value of 0.05 as a threshold for significance. All statistical analyses were performed using SPSS 17.0.

## Results

**Figure 1:**
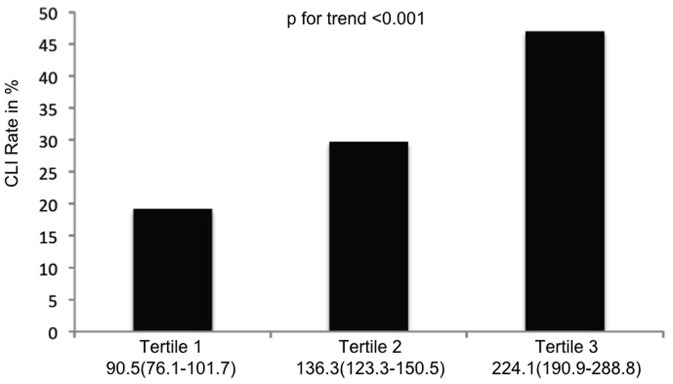
Percentage of patients with CLI stratified by tertiles of PLR. Numbers below the figure are median PLR and the 25^th^ and 75^th^ percentile.

A total of 2121 PAOD patients were included in the current analysis. Patientś characteristics are shown in Table 1. In a first step the study population was categorized according to the PLR into three tertiles each containing 707 patients. In the first tertile (median PLR 90.5, 76.1–101.7) the CLI rate was 19.2%, in the second tertile (median PLR 136.3, 123.3–150.5) the CLI rate was 29.7%, and in the third tertile (median PLR 224.1, 190.9–288.8) the CLI rate was 47.0% (Figure 1). In order to evaluate the trend for increase of CLI rate for increasing PLR a Jonckheere-Terpstra test was performed and showed statistical significance (p<0.001).

**Table 1:** Patientś characteristics of all PAOD patients included in the study.

n	2121
Age in years, median (25^th^–75^th^ percentile)	71(61–79)
Men, n(%)	1256(59.2)
BMI in kg/m^2^, median (25^th^–75^th^ percentile)	26(24–28)
**Vascular risk factors**	
Hypertension, n(%)	1727(81.3)
Type 2 diabetes, n(%)	720(33.9)
**Vascular endpoints**	
Prior myocardial infarction, n(%)	95(4.5)
TIA, prior stroke, n(%)	171(8.1)
**Concomitant disease**	
Atrial fibrillation, n(%)	373(17.6)
Congestive heart failure, n(%)	202(9.5)
Coronary artery disease, n(%)	752(35.4)
Cerebrovascular arterial disease, n(%)	1466(69.1)
Critical limb ischemia, n(%)	680(32.1)

In a second step a PLR value of 150 was calculated by receiver operating curve analysis as an optimal cut-off value to discriminate between CLI and non-CLI. Consequently, we categorized our cohort into two groups: one group with a PLR≤150 containing 1228 patients and a second group with a PLR>150 containing 893 patients. The first group contained 270(22.0%) CLI patients whereas the second group included 410(45.9%) patients with CLI. The difference between groups was statistically significant (p<0.001). Between the two PLR groups we found further statistically significant differences in other vascular endpoints (prior myocardial infarction (42(3.5%) vs. 51(5.7%), p = 0.02), in occurrence of ulcerations (209(17.0%) vs. 342(38.3%), p<0.001), and in inflammatory parameters (CRP (median 5.0 mg/l (2.0–10.0) vs. 7.0 mg/l (3.0–24.25) and fibrinogen (median 372 mg/dl (317.25–455.75) vs. 457.0 mg/dl (359.0–583.0); both p<0.001) as well (Table 2). Further differences between the two groups were found in platelet count (median 214.0 G/l (178.0–252.0) vs. 262.0 G/l (216.0–319.0), p<0.001) and also concerning hemoglobin (median 14.0 g/dl (12.9–14.9) vs. 12.5 g/dl (11.2–13.7), p<0.001). We further calculated differences in PLR between patients with and without ulcerations (median 170.8 (123.6–247.5) vs. 126.3 (96.5–173.8), p<0.001). We also did statistical analyses on the correlation of PLR with CRP and fibrinogen. We calculated a Pearson correlation and a Spearmańs rho as well for PLR>150 and CRP as for PLR>150 and fibrinogen. For both parameters, we could confirm a statistically highly significant correlation between the PLR and CRP/fibrinogen (p-value of <0.001; for PLR>150 and CRP: Pearsońs r = 0.3; Spearmańs rho = 0.2; for PLR>150 and fibrinogen: Pearsońs r = 0.4; Spearmańs rho = 0.3).

**Table 2:** Clinical and hematological characteristics of population with PLR≤150 and PLR>150.

	PLR≤150	PLR>150	P-value
n	1228	893	
Age in years, median (25^th^–75^th^ percentile)	68(59–76)	75(66–81)	**<0.001**
Men, n(%)	787(64.1)	469(52.5)	**<0.001**
BMI in kg/m^2^, median (25^th^–75^th^ percentile)	26(23–29)	25(22–28)	0.2
Hypertension, n(%)	984(80.0)	745(83.3)	0.06
Type 2 diabetes, n(%)	382(31.1)	338(37.8)	**0.001**
Prior myocardial infarction, n(%)	42(3.5)	51(5.7)	**0.02**
Atrial fibrillation, n(%)	160(13.1)	211(23.6)	**<0.001**
TIA, prior stroke, n(%)	87(7.1)	84(9.4)	0.06
Congestive heart failure, n(%)	80(6.6)	118(13.3)	**<0.001**
Coronary artery disease, n(%)	413(33.8)	333(37.4)	0.1
Cerebrovascular arterial disease, n(%)	687(67.4)	775(70.5)	0.1
Critical limb ischemia, n(%)	270(22.0)	410(45.9)	**<0.001**
Patients with ulceration, n(%)	209(17.0)	342(38.3)	**<0.001**
CRP in mg/l, median (25^th^–75^th^ percentile)	5.0(2.0–10.0)	7.0(3.0–24.25)	**<0.001**
Fibrinogen in mg/dl, median (25^th^–75^th^ percentile)	372.0(317.25–455.75)	457.0(359.0–583.0)	**<0.001**
Platelets in G/l, median (25^th^–75^th^ percentile)	214.0(178.0–252.0)	262.0(216.0–319.0)	**<0.001**
Hemoglobin in g/dl, median (25^th^–75^th^ percentile)	14.0(12.9–14.9)	12.5(11.2–13.7)	**<0.001**
PLR, median (25^th^–75^th^ percentile)	106.0(87.2–125.7)	204.2(172.7–265.8)	**<0.001**

In a third step PLR>150 was used as a variable in a binary logistic regression model to evaluate this value as an independent risk factor for CLI. In this model NLR>3.95, sex, type 2 diabetes, age>75, coexistence of congestive heart failure, history of stroke/TIA and the coexistence of atrial fibrillation were additionally included. NLR>3.95, type 2 diabetes and age>75 were included as these variables showed a close association with a coexisting CLI in studies published recently from our group [6], [11]. Even after adjustment for NLR>3.95, age>75, type 2 diabetes, sex, coexistence of congestive heart failure, history of stroke/TIA and atrial fibrillation PLR>150 was associated with an OR of 1.9 (95%CI 1.7–2.1, p<0.001) for CLI (Table 3).

**Table 3:** Adjusted risk factors for CLI in PAOD patients.

Risk factor	Adjusted odds ratio (95% CI)	P-value
PLR>150	1.9(1.7–2.1)	**<0.001**
NLR>3.95	1.7(1.5–1.9)	**<0.001**
Age ≥75 years	2.1(1.9–2.3)	**<0.001**
Sex	1.1(0.9–1.3)	0.4
Type 2 diabetes	2.7(2.5–2.9)	**<0.001**
Congestive heart failure	0.9(0.6–1.2)	0.6
TIA, prior stroke	1.2(0.8–1.6)	0.2
Atrial fibrillation	1.8(1.5–2.1)	**<0.001**

The factors were adjusted in a binary logistic regression model.

## Discussion

In this study we were able to demonstrate that a PLR>150 proved to be at least similar to NLR>3.95, an already published vascular risk factor, in its association with CLI in PAOD patients. Even after adjustment for other main CLI risk factors like NLR>3.95, diabetes and age >75 years PLR>150 was associated with a 1.9fold increase in CLI risk. However, not only CLI was more frequently found in the high PLR group. Endpoints due to atherosclerotic lesions in other vascular beds, like myocardial infarction, were also more frequent in this group. Even entities associated with coronary artery disease, like congestive heart failure and atrial fibrillation [12], were significantly more prevalent in the group with PLR>150.

Recently, the PLR has been published to enable the prediction of limb salvage in CLI patients [13]. By means of NLR and PLR CLI patients with a high risk for limb amputation during a five year follow up period were discriminated from patients with a lower risk for limb amputation during the same follow up period [13]. One possible reason for this finding could be a change in blood viscosity due to higher and lower platelet counts leading to higher and lower PLR. Platelets further increase in case of inflammation as could be found in patients with active atherosclerosis leading to a more aggressive course of their disease. This theory is also underlined by the fact that in our patients the median PLR was significantly higher in patients with ulcerations compared to those without ulcerations. Therefore not only an association of PLR with the outcome in CLI patients is obvious, but also an association of PLR and CLI itself in PAOD patients seems likely, as we were able to demonstrate by our data. In our study the PLR also significantly correlated with other inflammatory markers, namely CRP and fibrinogen, further underlining this hypothesis.

Platelets play an important role in the progression of atherosclerosis. According to current research platelets interact with endothelial cells and leukocytes [14] and release inflammatory substances leading to adhesion and transmigration of monocytes [15]. These monocytes support inflammatory processes in the vessel wall promoting atherosclerotic lesions [16]. An elevated platelet count leading to an elevated PLR might therefore lead to an increase in vascular endpoints. Interestingly in our data the occurrence of coronary artery disease did not differ significantly between patients with low PLR (≤150) and those with elevated PLR (>150), whereas the vascular endpoints as CLI and myocardial infarction differed significantly. This is in harsh contrast to the findings on NLR published recently from our group [6]. We were able to show that NLR>3.95 was associated with vascular endpoints as well as it was associated with atherosclerotic lesions. The NLR seems to reflect the inflammation in the vessel wall, whereas the PLR seems to reflect the endpoint also caused by higher blood viscosity. This finding is also underlined by the fact that in our regression model the PLR was superior to the NLR in its association with CLI.

Recently not only platelets but also microparticles (MP) derived from platelets were postulated to be associated with formation and progression of arterial thrombi in the context of manifest atherosclerosis and following injury of the vessel [8]. A key feature of this platelets derived MPs is the procoagulant activity enabling them a central role in haemostasis and thrombosis. An elevated PLR might also enhance MP production leading to a state of activated haemostasis leading to an increase of CLI and other vascular endpoints, like myocardial infarction, as we were able to find in our patients.

The second constituent of the PLR besides platelets is the lymphocyte-count. Especially in CLI lymphocytes seem to play an important role in the clinical course of the disease. Iso et al. investigated the role of implanted bone marrow cell composition on limb salvage in CLI patients [17]. In this study lymphocytes were significantly elevated in the limb salvage group [17]. One possible explanation for this finding is that lymphocytes might also be associated with the mediation of collateral growth via IL-16 secretion. This was shown recently in a murine hindlimb ischemia model [18]. This might also be an explanation for our findings, as patients with a high lymphocyte count, leading to a lower PLR might have more collateral growth leading to less ischemia and therefore less CLI.

Similar to other studies conducted in this field our patients in the high PLR group were significantly older than the patients in the low PLR group. Whether age influences the PLR has to our knowledge not been investigated in the literature so far. However, the association of an elevated PLR with CLI was still significant in our patients even after adjustment for the factor age>75 years.

In a recent study we showed that an elevated CHA_2_DS_2_-VASc Score is associated with an elevated CLI risk [11]. By means of this scoring system PAOD patients with a high CLI risk can be discriminated from those with a low CLI risk. Especially diabetes and age>75 years, both factors associated with mediasclerosis in the literature, were the two main entities associated with an elevated CLI-increase [11]. In our current study we were able to show that a PLR>150 was associated with an OR of 1.9 for CLI, even after adjustment for these two main CLI risk factors. Similar to diabetes and the age of the patients, which can both be obtained from the medical history, the PLR is an easy available test and can be calculated from the whole blood count. The whole blood count is a laboratory test, which is usually performed on admission to the hospital in most patients.

The main drawback of our study is the retrospective study design and that we used a single blood sample to calculate PLR. It therefore remains unclear whether this single blood sample reflects an elevated PLR over time. Furthermore for exact stratification of the impact of PLR on future vascular events prospective studies are needed. In these studies also differences between PLR in acute and chronic PAOD patients can be evaluated. As various parameters, like CRP, diabetes, and age differed significantly between our patients with high and low PLR these parameters might affect our results although we adjusted these results for confounding factors in a regression analysis.

However, we were able to show that PLR>150 can be used to discriminate patients at high risk for CLI from those with a low CLI-risk. Especially in combination with NLR, diabetes and the age of the patients a discrimination of PAOD patients with high CLI risk seems possible.
